# Focus on UV-Induced DNA Damage and Repair—Disease Relevance and Protective Strategies

**DOI:** 10.3390/ijms21197264

**Published:** 2020-10-01

**Authors:** Mateusz Kciuk, Beata Marciniak, Mariusz Mojzych, Renata Kontek

**Affiliations:** 1 Doctoral School of Exact and Natural Sciences, University of Lodz, Banacha Street 12/16, 90-237 Lodz, Poland; 2 Department of Molecular Biotechnology and Genetics, Faculty of Biology and Environmental Protection, University of Lodz, 12/16 Banacha St., 90-237 Lodz, Poland; beata.marciniak@biol.uni.lodz.pl (B.M.); renata.kontek@biol.uni.lodz.pl (R.K.); 3 Department of Chemistry, Siedlce University of Natural Sciences and Humanities, 3 Maja 54, 08-110 Siedlce, Poland; mmojzych@yahoo.com

**Keywords:** UV radiation, DNA damage, DNA repair, photoproducts, ROS

## Abstract

The protective ozone layer is continually depleting due to the release of deteriorating environmental pollutants. The diminished ozone layer contributes to excessive exposure of cells to ultraviolet (UV) radiation. This leads to various cellular responses utilized to restore the homeostasis of exposed cells. DNA is the primary chromophore of the cells that absorbs sunlight energy. Exposure of genomic DNA to UV light leads to the formation of multitude of types of damage (depending on wavelength and exposure time) that are removed by effectively working repair pathways. The aim of this review is to summarize current knowledge considering cellular response to UV radiation with special focus on DNA damage and repair and to give a comprehensive insight for new researchers in this field. We also highlight most important future prospects considering application of the progressing knowledge of UV response for the clinical control of diverse pathologies.

## 1. Introduction

The ozone layer in the upper atmosphere is continually diminished due to release of pollutants that damage the protective barrier. As a consequence, exposure to ultraviolet (UV) light is increasing. DNA is the main target of UV radiation both in lower organisms such as bacteria and higher such as placental mammals including humans [1,2]. Deteriorating effects of UV radiation on DNA include formation of various photoproducts, generation of free radicals and subsequent introduction of strand breaks [3]. In response to these threats, cells have evolved multiple mechanisms that counter the damage. The DNA damage response (DDR) includes multitude damage signaling pathways and repair mechanisms that allow the preservation of genomic stability of cells [4,5].

## 2. UV-Induced DNA Damage

**Figure 1:**
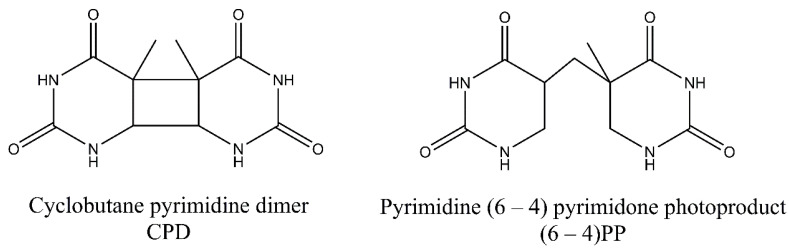
Common ultraviolet (UV)-induced photoproducts. Cyclobutane-pyrimidine dimers (CPDs) comprise 75% of photodamage, while 6-4 photoproducts (6-4PPs) constitute 25% of photodamage. Derivatives of two thymine bases were shown in the figure.

The two main photoproducts that arise during exposure to UV light include cyclobutane-pyrimidine dimers (CPDs), and 6-4 photoproducts (6-4PPs), but less prevalent Dewar valence isomers also may arise (Figure 1). These less common photoproducts emerge when an additional photon is absorbed by (6-4PPs) [6]. DNA is damaged predominantly by longer wavelength radiation—UV-B (280–315 nm) and UV-A (315–400 nm)—while UV-C (<280 nm) is considered less harmful as it is absorbed by oxygen and the ozone layer of the atmosphere. UV-A, on the other hand, is a much less deleterious damaging factor than UV-B because it is not so effectively absorbed by the DNA. However, it can act as a photosensitizer and can promote the formation of secondary photoproducts [1,7]. The energy content of UV-radiation decreases with increase in wavelength with order of: UV-C, UV-B and UV-A. The aromatic ring structures of DNA bases absorb short wavelength UV more efficiently, thus UV-C and UV-B is effectively captured [8]. Moreover, longer UV wavelengths may also induce small amounts of DNA breaks and DNA-protein and interstrand cross-links [9,10,11,12,13]. 

Despite the fact that 6-4PPs cause more prominent perturbations in the structure of DNA helix, CPDs are the main cytotoxic lesions responsible for cell death following UV exposure. However, some studies indicate that, due to inability of cells to bypass 6-4PPs, this photolesion may constitute an important contributor to cell death [14]. In contrast, some studies indicate that the formation of 6-4PPs may have no impact on mutagenesis [15]. Also, 6-4PPs may undergo photoisomerization to Dewar isomers upon exposure to UV-B or UV-A. This reaction is thought to be efficiently catalyzed by the wavelengths longer than 290 nm [1]. Moreover, CPDs may also be reverted upon exposure to UV-light of 230 nm wavelength [15].

## 3. Pyrimidine Photoproducts

In CPDs adjacent pyrimidine bases are covalently linked in [2 + 2] cycloaddition reaction involving C5 and C6 carbon atoms, while in 6-4PPs, a noncyclic bond is formed between the C6 and C4 atoms of pyrimidine residues. The formation of 6-4PPs is a two-step process in which two intermediates can be formed. The oxetane intermediate is formed when the 3′-end is thymine. On the other hand, an azetidine intermediate is formed when the 3′-end is cytosine. For CPDs two bases can be on the same or on the opposite sides of cyclobutane ring. These stereoisomers are referred to as cis and trans stereoisomers. Additionally, bounding of C5 (or C6) atom of one pyrimidine with the C5 (or C6) atom of the second pyrimidine leads to syn configuration of the bases. The anti-configuration is achieved when the C5 atom of one pyrimidine is linked with the C6 atom of other pyrimidine base. It has been found that cis-syn-configured CPDs are formed much more frequently than trans-syn-configured CPD. CPDs may also be reverted to original bases following exposure to UV-C light [2,7,16]. On the other hand, 6-4PPs exist only in two stereoisomers after irradiation. Moreover, 6-4PPs may be converted into Dewar valence isomers. The new covalent bond is then formed between the N3 and C6 atoms of the pyrimidone following absorption at 315/325 nm. For both photoproducts containing cytosine bases, a deamination reaction may occur. In this reaction, an exocyclic amine group in a cytosine is lost, and the base is converted into uracil [16]. The formed uracil can be further converted to thymine after two rounds of DNA replication. Additionally, cytosine residues in CpG sequences can be modified to 5-methylcytosine and deaminated to thymine residues [17].

**Figure 2:**
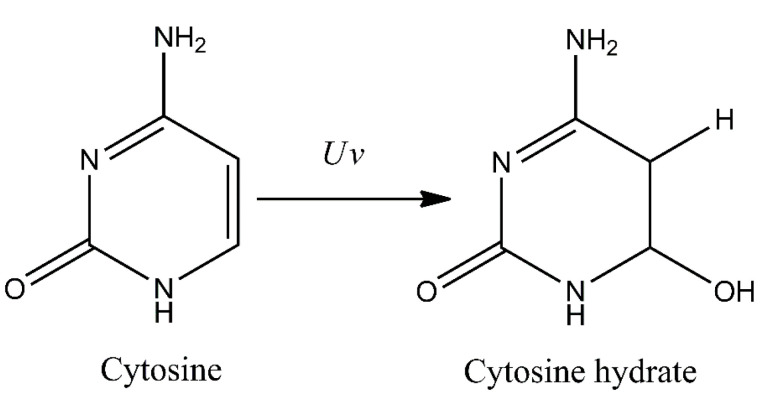
Formation of cytosine photohydrate (6-hydroxy-5,6-dihydrocytosine) as a result of photohydration reaction.

Additionally, several other minor photoproducts were identified. These include cytosine photohydrate (6-hydroxy-5,6-dihydrocytosine) formed during addition of water molecule to a C5–C6 double bond of the cytosine (photohydration reaction) (Figure 2). However, cytosine hydrate and its stereoisomers were found to be very unstable and represent only minor photoproducts [18,19,20].

## 4. Purine Photoproducts

**Figure 3:**
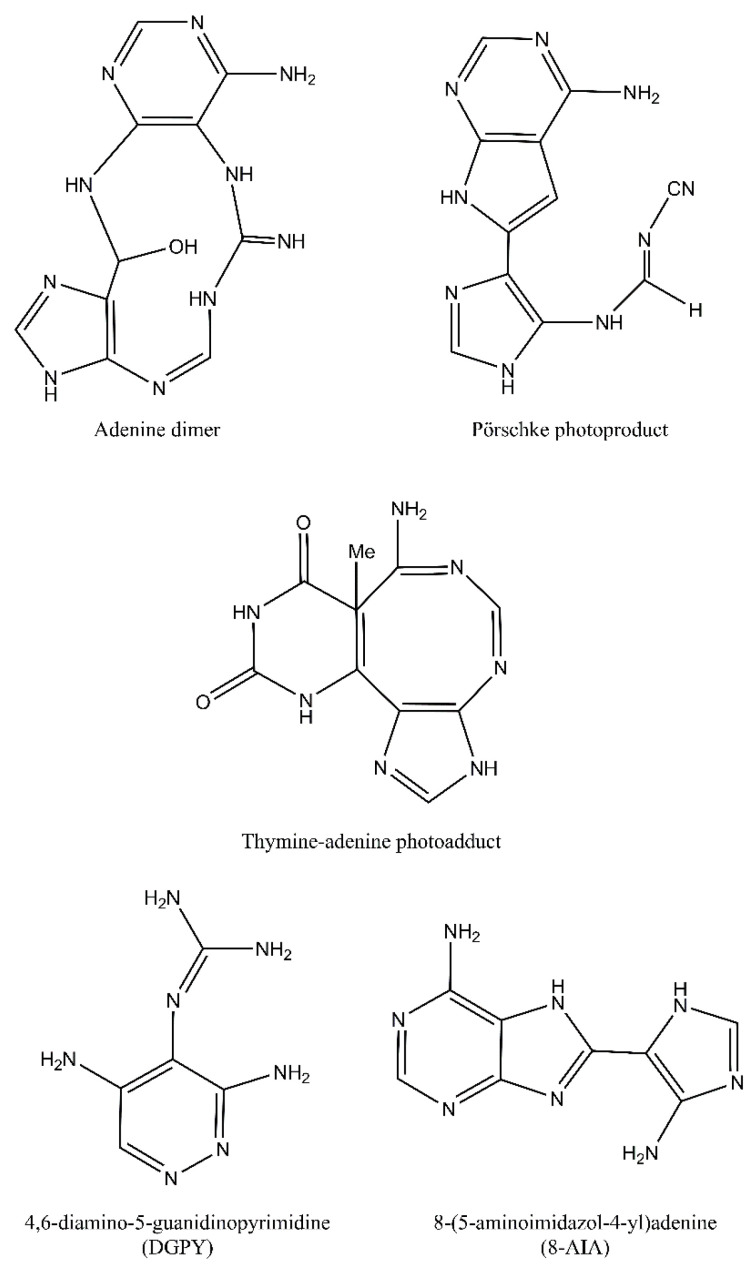
Purine photoproducts: adenine dimer, Pörschke photoproduct, thymine-adenine photoadduct, 4,6-diamino-5-guanidinopyrimidine (DGPY), and 8-(5-aminoimidazol-4-yl)adenine (8-AIA).

Despite the fact that pyrimidine photoproducts are the predominant lesions induced by UV radiation, adenine-thymine photodimers may also arise. Following cycloaddition of the N7-C8 double bond of the 5′-A across C6 and C5 positions of the 3′-A, an azetidine intermediate is formed. This intermediate molecule is a base for two adenine photoproducts: adenine dimers and Pörschke photoproducts. These two photoproducts may then be converted to 4,6-diamino-5-guanidinopyrimidine (DGPY) and 8-(5-aminoimidazol-4-yl)adenine (8-AIA). Chemical structures of purine photoproducts are presented in Figure 3 [7].

## 5. UV-Associated ROS Damage

**Figure 4:**
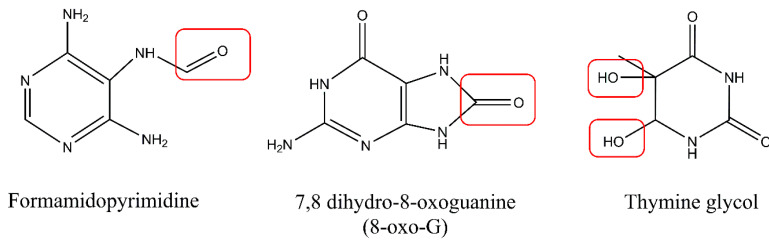
Oxidized DNA bases: formamidopyrimidine derivative of adenine (Fapy-A), 7,8 dihydro-8-oxoguanine (8-oxo-G), and thymine glycol. Modifications of the proper base structures by ROS were highlighted with the red boxes.

UV light may also indirectly cause DNA damage via production of singlet oxygen or free radicals in photodynamic reactions. One of the most reactive oxygen species (ROS) is hydroxyl radical (OH*), with its high DNA damaging capacity [21]. The main mechanism by which DNA can be damaged by ROS is the generation of oxidized nitrogen bases. Approximately 100 oxidatively generated bases and 2-deoxyribose modifications have been identified so far [22]. Oxidation of pyrimidines results in the formation of hydrates and glycol derivatives, while oxidation of purines leads to production of formamidopyrimidine derivatives, 8-oxo derivatives (7,8 dihydro-8-oxoguanine), and 2-hydroxyadenine (Figure 4). Guanine is especially prone to oxidation and poses a threat to genomic stability. Also, 8-oxoguaniane may alter DNA’s structure and lead to disruption of both the replication and transcription processes. In addition to causing base damage, ROS may also contribute to the formation of single strand breaks (SSBs) in DNA via direct attack on the sugar phosphate backbone of the DNA molecule. Arising on both strands, single-strand breaks may be converted into more lethal double-strand breaks (DSBs). Additionally, it was observed that DSBs may in fact be formed during replication of unrepaired photoproducts during replication fork arrest. Despite causing DNA damage, ROS can also damage other molecules present in cells, such as lipids and proteins. In a process of lipid peroxidation, aldehydes such as malondialdehyde or 4-hydroxynonenal can be formed. These reactive aldehydes can form DNA adducts with adenine, cytosine, or guanine [23,24,25]. Furthermore, work by Strumberg et al. [26] has shown that the inhibition of topoisomerase I may result from exposure to UV light and lead to the formation of topoisomerase I cleavage complexes, which can be further converted into DSBs. This indicates that, following UV-induced replication stress, some DSBs may be formed in a topoisomerase I-dependent manner via religation blockage [26]. Similarly, ROS induction may lead to abortive activity of topoisomerase II that cause DSBs formation [27,28]. Excellent reviews on the formation of ROS-induced DNA damage exist [15,24,29].

## 6. Factors That Affect Formation of UV Damage

The main factors that contribute to the formation of certain type of lesions are (a) the chemical nature of base involved, (b) the sequence of bases in DNA molecule (the G:C content), (c) the topography and conformation of DNA (lower yields of photoproducts after denaturation of DNA helix), (d) binding of other factors that influence chromatin condensation status, and (e) the radiation wavelength. One of the critical factors that influence both damage occurrence and repair capacity is certainly the chromatin condensation status and existing chromatin dynamics. Nucleosome represents the basic repeating unit of chromatin. It consists of a core built of conserved histone proteins H2A, H2B, H3, and H4 that form an octamer and bind a ~147-bp-long fragment of the DNA molecule. Additionally, the coiling of DNA is affected by the linker histones that are bound to the linker DNA between adjacent octamers. Electrostatic interactions of histone proteins and DNA strongly influence the binding strength and accessibility of DNA along the sequence. The binding strength may be further influenced by (a) exchange of histone variants, (b) ATP-dependent chromatin remodeling, and (c) post-translational modifications of the core histones. To what extent these factors influence the emergence of UV damage remains to be elucidated [30,31,32,33,34,35].

Within the nucleosome core, the rotational and translational changes of DNA sites may occur [36]. The unwrapping and rewrapping of DNA into the histone core is mainly dependent on DNA sequence [37,38]. T-T and T-C CPDs are formed more frequently than C-T and C-C counterparts. Nucleosomes play the pivotal role in protecting those T-T rich sequences from UV light [1,16]. The GC% in the DNA is crucial factor that impacts the amount of T-T and C-photoproducts. The higher the GC content of the DNA, the lower the amount of T-T photoproducts generated. In contrast to CPDs, T-C 6-4PPs are formed in DNA much more frequently than TT 6-4PPs [16]. 

Studies indicate that CPDs have more random distribution between linker DNA and the core of the chromatin, while 6-4PPs mainly arise in the linker regions. Moreover, CPDs form more promptly where the phosphate backbone is furthest from the histone core, while 6-4PPs have more random distribution within nucleosome [39]. Various studies have shown that bending or unwinding of DNA molecule is damage promoting factor [7]. UV photoproducts promote DNA unwrapping from the histone octamers and facilitate access of repair proteins to the site of damage. However, studies in yeast indicate that rotational setting does not influence NER activity and suggest that it only determines CPD formation. Additionally, CPDs formed in distal regions of nucleosomal DNA are repaired more promptly. This is concurrent with the studies that indicate that unwrapping occurs more quickly in such distal regions. Furthermore, binding of certain transcription factors (TF) to DNA may modulate the formation of CPDs and repair capacity [39]. However, damage formation is highly individual and dependent on the TF involved, the type of UV damage, and its position in the DNA sequence. The variation is attributed mainly to structural changes of DNA upon TF binding [40]. 

As previously mentioned, chromatin status plays an important role in modulation of cellular capacity to remove damage. This process involves deposition of newly synthesized histones such as H2A, H3.1, and H3.3 at damage sites by specific histone chaperone proteins: acidic leucine-rich nuclear phosphoprotein 32 family member E (ANP32E), chromatin assembly factor 1 (CAF-1), facilitates chromatin transcription (FACT), or histone regulator A (HIRA) [41]. ANP32E removes H2A.Z histone from damaged chromatin encouraging chromatin accessibility. FACT, on the other hand, is responsible for H2A-H2B turnover and promotes deposition of γH2AX (phosphorylated on serine 139 histone H2AX) contributing to damage signaling. However, the issue of repair process in the context of chromatin is not the main objective of the article. Thus, we encourage further reading on this topic [42,43,44,45].

## 7. Cellular Response to UV Radiation

### 7.1. Kinase Signaling Pathways

Cells constantly communicate through a variety of signaling pathways. These signaling dialogs involve activation of mitogen-activated protein kinase (MAPK) including the extracellular signal-regulated kinases (ERKs), the c-Jun NH2-terminal kinases (JNKs), and the p38 kinases); epidermal growth factor receptor (EGFR) pathway; atypical protein kinase C’s (aPKCs): PKC-λ/ι and PKC-ζ pathways; phosphoinositide 3-kinase-protein kinase B (PI3K-AKT) pathway; or the tumor necrosis factor α (TNFα) pathways previously reviewed by references [46,47,48,49]. The induction of signaling pathways depends on several factors including dose, exposure time, and wavelength of UV radiation [46]. ROS generated in response to UV radiation may act as activators of signal transducing pathways. However, to what extent this occurs remains to be elucidated. Moreover, a multitude of cytokines can be induced following UV exposure, and their role in signaling pathways is of particular importance [47]. 

### 7.2. Transcriptional Response to UV Radiation

The signaling cascades usually lead to alterations in gene transcription. Upon UV irradiation, certain genes involved in proliferation and apoptosis become activated (up-regulated) or silenced (down-regulated). This is mainly attributed to DNA-binding proteins or transcription factors activated in signaling cascades. Two earliest identified transcription factors activated in response to UV radiation are activating protein 1/2 (AP-1/2) and nuclear factor kappa B (NF-κB). However, nuclear factor of activated T cells (NFAT), signal transducers and activators of transcription (STATs), and cellular tumor antigen p53 (TP53) may also take part in the UV response [8,47,50,51,52]. TP53 also works as an important factor that regulate chromatin accessibility allowing relaxation of the chromatin upon UV damage [53]. Moreover, computational studies indicated 832 and 1236 up- or downregulated genes in response to UV radiation, respectively. In UV-treated mouse fibroblasts, this response is connected with enhanced translational response (upregulation of genes involved in translation initiation or elongation), upregulation of genes responsible for oxidation reduction, stress response, and apoptosis. In contrast, many more genes were shown to be downregulated. These included genes involved in transcriptional regulation, chromatin organization, biosynthesis and proteolysis. Interestingly, a significant reduction in the accessibility of chromatin following UV radiation was suggested. This may indicate the protective role of chromatin compaction against further damage. However, at the same time, it may limit the accessibility of repair proteins to the sites of damage impeding its removal. Moreover, the down-regulation of genes presented in the study may be the result of overall elevated condensation of chromatin following UV exposure [45]. Studies in cell lines from 15 individuals also indicate a wide array of transcriptional changes in response to UV irradiation. Similarly to the results of computational studies, number of genes involved in cell cycle inhibition (such as p21, *TOB1*, *CCNG1*, *BTG1*, *RARRES*, *S100A11*, and *RUNX3*) and progression (*MCM6*, *NOLC1*, *PTMA*, *DP1*, *DUSP5*, *POLE3*, *SRM*, *ODC*, and *AMD1*) were upregulated following UV treatment. The full names of the genes are placed in the abbreviations section for clarity. Moreover, *BAX*- and *TNF*-related genes were the prominent significantly up-regulated apoptotic genes, while *PEA15*, *DDR1*, *TRAF1*, *BIRC3*, *IER3*, and *TAX1BP1* were the most highly expressed anti-apoptotic genes. UV radiation induced both genomic nucleotide excision repair—NER (DDB2, XPC, GADD45, and PCNA) and transcription-coupled repair genes (CSA and CSB). Additionally, O6-methylguanine-DNA methyltransferase (MGMT) involved in the repair of alkylation damage in human cells was shown to be downregulated following UV treatment. It is, however, unknown how the cells coordinate the expression of the exact genes in response to UV radiation [54] 

### 7.3. Canonical DNA Damage Response and UV Radiation

In response to UV radiation, a cell elicits DDR as an effective way to signal and repair the emerging damage. Serine-protein kinases ataxia telangiectasia mutated (ATM) and ataxia telangiectasia and Rad3-related protein (ATR) together with DNA-dependent protein kinase catalytic subunit (DNA-PKcs) and poly [ADP-ribose] polymerases (PARPs) comprise a central part of the signaling hub activated in response to various genetic insults. ATM and DNA-PKcs are activated mainly in response to double-strand breaks, while single-strand breaks and other damage associated with the replication process are the ATR-activating factors. The regulation of the damage signaling pathways results from direct protein-protein interactions or post-translational modifications. Moreover, aforementioned kinases modulate the structure of chromatin near DNA damage and provide an appropriate cellular environment for the effective repair of the damage [55,56]. 

**Figure 5:**
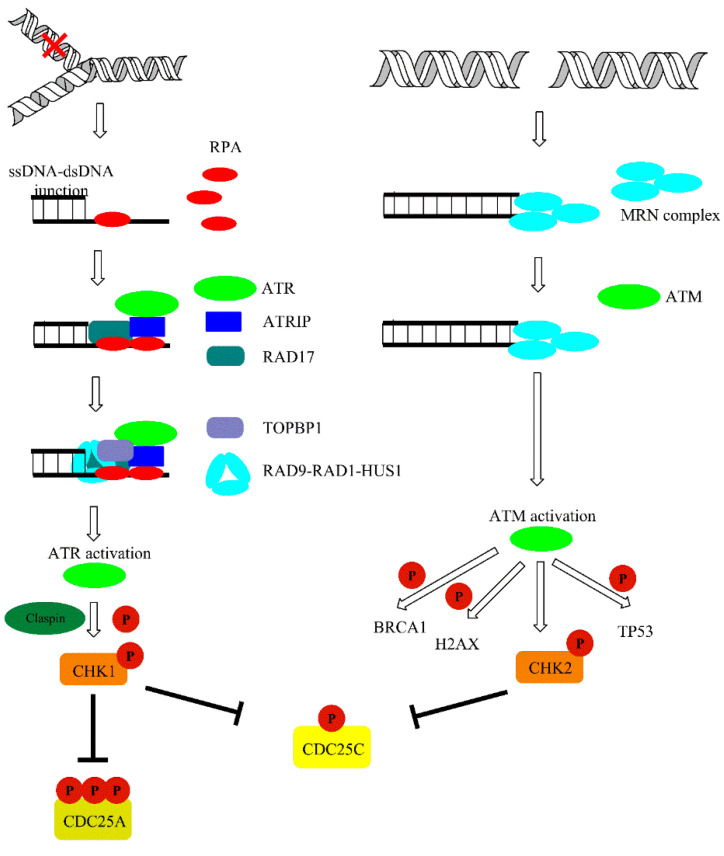
Ataxia telangiectasia and Rad3-related protein (ATR) and ataxia telangiectasia mutated (ATM)-mediated DNA damage response (DDR) pathways activated upon DNA damage. The details are discussed in text.

DNA damage is detected by MRE11-RAD50-NBS1 (MRN) protein complexes. Recruitment of former proteins to the damage sites aim to transmit the information about damage to ATM kinase [57]. Both ATM and ATR exist as inactive dimers and monomerize in the activation process. Oxidation may also directly activate ATM, independently of MRN complex. ATM phosphorylates a variety of substrates following DNA damage, including acting as a mediator of DNA damage checkpoint protein 1 (MDC1), nibrin (NBS1), TP53-binding protein 1 (53BP1), and breast cancer type 1 susceptibility protein (BRCA1). However, a primer and one of the most important substrate of both ATM and ATR kinase is certainly histone protein H2AX. Phosphorylation of H2AX at serine 139 leads to γH2AX foci formation at damage sites. Furthermore, γH2AX foci are responsible for the recruitment of MDC-1, which works as a platform or mediator for other DDR proteins and amplifies the signaling pathway via recruitment of other DDR components. In addition to phosphorylation, DDR is coordinated by E3 ubiquitin-protein ligase (RNF8 and RNF168)-mediated ubiquitylation, which leads to the accumulation of 53BPI, BRCA1, and other proteins at the site of damage. Furthermore, it may affect the choice between homologous recombination (HR) and non-homologous end joining (NHEJ) repair pathways. Other than RNF8/168, ubiquitin-protein ligases may also take part in DDR. For example, the RNF20-RNF40 heterodimer monoubiquitylates H2B histone in proximity to DSB affecting chromatin condensation status for effective damage removal. Additionally, γH2AX affects chromatin status via recruitment of chromatin remodeling complexes such as SWI/SNF, SWR1, and chromatin-remodeling ATPase INO80 (INO80) to chromatin in response to damage occurrence. Moreover, it attracts other DDR factors and is crucial for the repair processes. ATM may also phosphorylate other factors responsible for chromatin compaction, such as nuclear corepressor KAP1 (KAP1). Thus, DDR is crucial for the modulation of chromatin dynamics following damage [56,58]. Single-stranded DNA that accumulates as a result of DNA replication licensing factor 7 MCM2-7 complex helicase activity is coated with replication protein A (RPA), interacts with ATR-interacting protein (ATRIP), and recruits ATR kinase to the sites of damage at stalled replication forks. Activation of ATR is dependent on DNA topoisomerase 2-binding protein 1 (TOPBP1) and cell cycle checkpoint control protein complex (RAD9-RAD1-HUS1) [57,59]. ATM and ATR activation leads to phosphorylation of downstream signal transducers, including serine/threonine-protein kinases (CHK1/2), and results in subsequent inhibition of cyclin-dependent kinases (CDKs) that slows down or halts the cell cycle in the G1-S, S, or G2-M phase of the cell cycle (Figure 5). 

**Figure 6:**
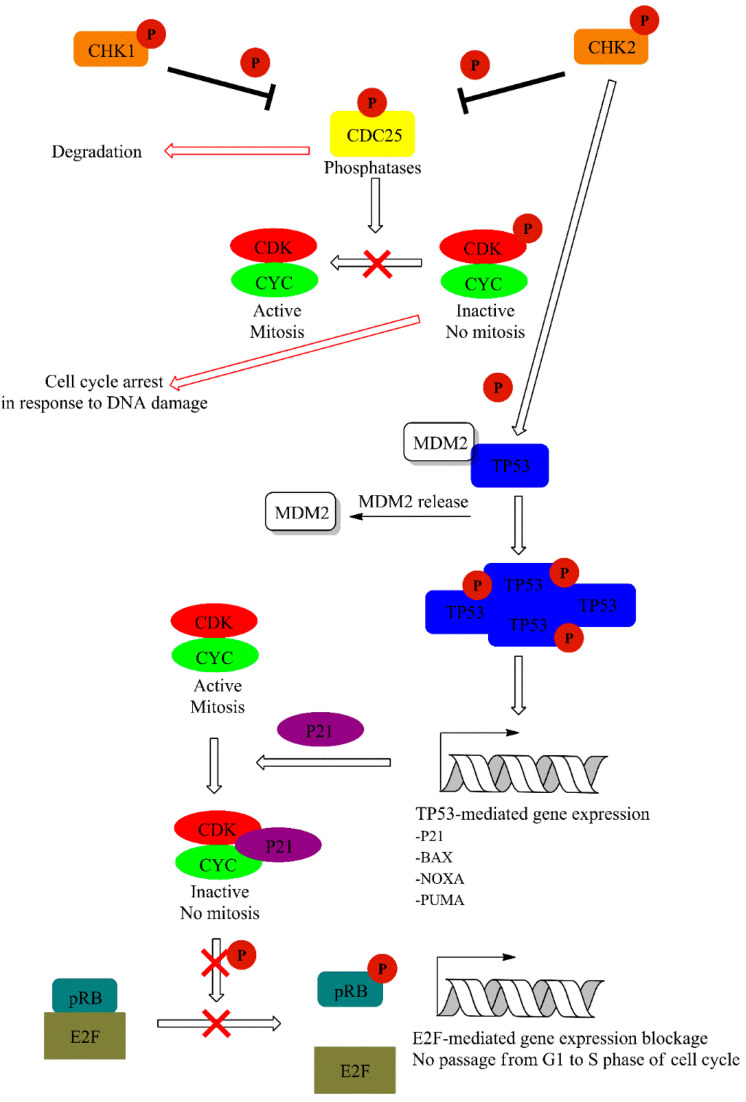
Downstream signaling cascade activated by ATR and ATM via CHK1/2 dependent phosphorylation events. A detailed description of the pathway can be found in the text.

CHK1 kinase is activated mainly by ATR, and this is mediated by Claspin protein. CHK1 regulates activity of two M-phase inducer phosphatases CDC25C and CDC25A. Under normal conditions, CDC25 phosphatases activate CDK1-cyklin B complexes, leading to cell cycle progression. Activation of CHK1 in response to DNA damage leads to phosphorylation and subsequent degradation of CDC25. This event leads to cell cycle arrest. On the other hand, CHK2 activation is mainly achieved via ATM-mediated phosphorylation and leads to TP53 activation. Additionally, ATM and ATR may also directly phosphorylate TP53 [60]. This is accompanied with the release of TP53 inhibitor—E3 ubiquitin-protein ligase (MDM2) [61]. TP53 is one of the best known suppressor proteins. It is estimated that half of all human tumors carry a mutation in the TP53 gene [62]. Phosphorylated in response to UV damage, TP53 forms tetrameric transcription factor that regulate expression of various genes including cyclin-dependent kinase inhibitor 1A (P21) and the pro-apoptotic (BAX) and Bcl-2-binding component 3 (PUMA) proteins. P21 (up-regulated in a TP53-mediated gene expression) form complexes with cyclin-CDK leading to cell cycle arrest. Furthermore, P21 inhibits CDKs preventing the phosphorylation of the retinoblastoma-associated protein (pRB), thereby disabling the G1 to the S transition of the cell cycle (Figure 6) [61,63,64].

BAX protein with Bcl-2 homologous antagonist/killer (BAK) form pores in mitochondrial membranes and are responsible for the release of cytochrome C from the mitochondria. As a consequence, activation of the intrinsic apoptotic cell death pathway occurs. The activity of BAX and BAK is restricted by anti-apoptotic proteins such as apoptosis regulator (BCL-2) or Bcl-2-like protein 1 (BCL-Xl). PUMA works as an apoptosis inducer because of the higher affinity of binding toward anti-apoptotic proteins. This property allows BAX and BAK embedment into the mitochondrial membrane [65].

Structurally different from ATM and ATR, DNA-PKcs are activated via direct interaction with KU70/80 protein and work together in NHEJ. Furthermore, DNA-PKcs recruit Artemis endonuclease and phosphorylates DNA ligase IV, allowing the ligation of the broken ends [61].

In contrast, poly [ADP-ribose] polymerases (PARPs) work as signaling molecules in single-strand break repair. PARPs catalyze an evolutionary conserved posttranslational modification in which ADP-ribose residues from nicotinamide adenine dinucleotide (NAD+) molecules are transferred onto amino acid moieties of various proteins, including histone proteins and PARP itself, leading to polymeric ADP-ribose chains formation. PARP1 and PARP2 are the main enzymes with a function in damage signaling. In fact, PARP1 is responsible for the majority of ADP-ribosylation events in cell. Poly-ADP-ribose chains act as a scaffold for repair proteins, ensuring effective recruitment of single-strand break repair (SSBR) and double-strand break repair (DSBR) machinery. PARP binding is only temporary due to emerging electrostatic interactions. Poly-ADP-ribose (PAR) chains are rapidly disassembled by poly (ADP-ribose) glycohydrolase (PARG), allowing the whole event to repeat [55,66]. 

PARP1 is directly recruited to UV-lesion sites following local irradiation [67]. UV radiation produces significant DNA damage that leads to excessive PARP-1 activation and cell death. However, PARP activation is crucial for damage signaling and only after achieving a particular threshold does cell death occur. Inhibition of PARP-1 with PARP inhibiting agents (such as low concentration of arsenite) may promote cell survival in response to UV radiation, but the cells most probably accommodate unrepaired DNA lesions contributing to UV-induced carcinogenesis [68]. Furthermore, inhibition or depletion of PARP1 significantly deteriorates UV-damage removal in human skin fibroblasts and epidermis. This is attributed to reduced nucleotide excision repair (NER) capacity in the absence of PARP1. The reduced NER activity may result from the lack of interaction between key lesion recognizer—damaged DNA-binding protein 2 (DDB2) or xeroderma pigmentosum group C (XPC) and PARP1 in the presence of UV damage affecting damage recognition [69]. Furthermore, DDB2 may stimulate catalytic activity of PARP-1 in the presence of UV-damaged DNA [67].

The activated signaling cascade finally leads to various cellular responses such as DNA repair, chromatin modelling, transcription, autophagy, senescence, and apoptosis. The type of DNA damage, its severity, and specific threshold determines the final response. ATM and ATR phosphorylate serine or threonine residues in the context of glutamine at the +1 position (the SQ/TQ motif). Thus, studies aimed to identify potential substrates of DDR kinases in response to UV radiation. What they found was the potential 216 SQ/TQ sites that were phosphorylated in UV-damaged cells. Furthermore, the study confirmed 39 sites more highly phosphorylated in UV-damaged ATR-wild type cells compared UV-damaged ATR-deficient cells. This gave a total of 231 SQ/TQ sites induced by UV damage dependent on ATR or both. The damage response to UV radiation involved multiple versatile proteins including those involved in cell cycle progression, chromatin binding, transcriptional regulation, DNA repair, and many other functions considered not typical for the damage response [70].

### 7.4. Cell Cycle Regulation in Response to UV-Induced Bulky DNA Adducts

Cell cycle regulation has an essential role in maintenance of the genome integrity. Its progression is tightly controlled by temporarily activated and deactivated serine/threonine kinases, number of mediators, and/or effectors [71]. Exposure to UV radiation may disturb proper cell cycle progression. Of many checkpoints, three are the most important—G1-S, intra-S, and G2-M. The G1-S and intra-S checkpoints prevent the replication of damaged DNA, while the G2-M checkpoint protects against segregation of damaged DNA to daughter cells [60]. As shown by previous research, UV radiation induces both G1 and G2 cell cycle arrest (in a p53-dependent or independent pathway, respectively) [72]. In cases of UV damage, ATR kinase plays a key role in the initiation of subsequent events such as the phosphorylation of p53 (G1-S) or Chk1(G2/M), as was previously mentioned in Section 7.3) [60]. 

Some reports indicate that other proteins, classical components of the DNA repair pathways, may be involved in cell cycle regulation upon occurrence of UV damage. These include proteins of the NER system, such as XPA and XPD, that are thought to have a dualistic nature and were shown to affect cell cycle checkpoint signaling [73,74]. The product of the *XPA* gene is the protein responsible for damage verification. Mutations in *XPA* have propounding effect on cellular physiology as the protein participates in both GG-NER and TC-NER. Moreover, defects in the XPA protein are responsible for the onset of Xeroderma pigmentosum (XP) with extreme sensitivity to UV radiation and higher predisposition to cancer [75]. Studies using XPA-deficient cells showed defects in ATR signaling upon DNA damage and suggest an additional role of XPA (to that observed in NER pathway) in damage signaling [73]. In contrast, the overexpression of XPD was shown to induce cell cycle arrest in G1 phase [74]. Another regulatory protein—DNA replication factor Cdt1 (CDT1)—seems to be involved in the formation of the pre-replication complex by replication “licensing” in the late M phase or early G1 phase. Morino et al. observed CDT1 degradation in M phase upon UV irradiation. As a consequence the “license” is blocked after the cell enters into the G1 phase of cell cycle (G1 arrest occurs) [76]. In summary defects in the DDR components may lead to malfunctioning of cell-cycle checkpoint and enhance the genomic instability of defective cells [7].

## 8. UV Damage Repair

### 8.1. Direct Reversal of Damage (DR)

The simplest example of DNA repair mechanisms is certainly direct reversal of damage. The main features of this damage control are (I) extreme simplicity manifested by participation of only one enzyme for each repair mechanism, (II) narrow substrate specificity and high accuracy, (III) and relation to previous points relatively high speed of action compared to multistep processes. There are three major types of direct DNA repair: (a) reversal of UV-induced photolesions carried by photolyases, (b) repair of O-alkylated DNA by O6-alkylguanine-DNA alkyltransferases, and (c) repair of N-alkylated bases by AlkB family dioxygenase enzymes [77]. Here, we focus on photolyases as the main enzymes responsible for UV-damage removal.

**Figure 7:**
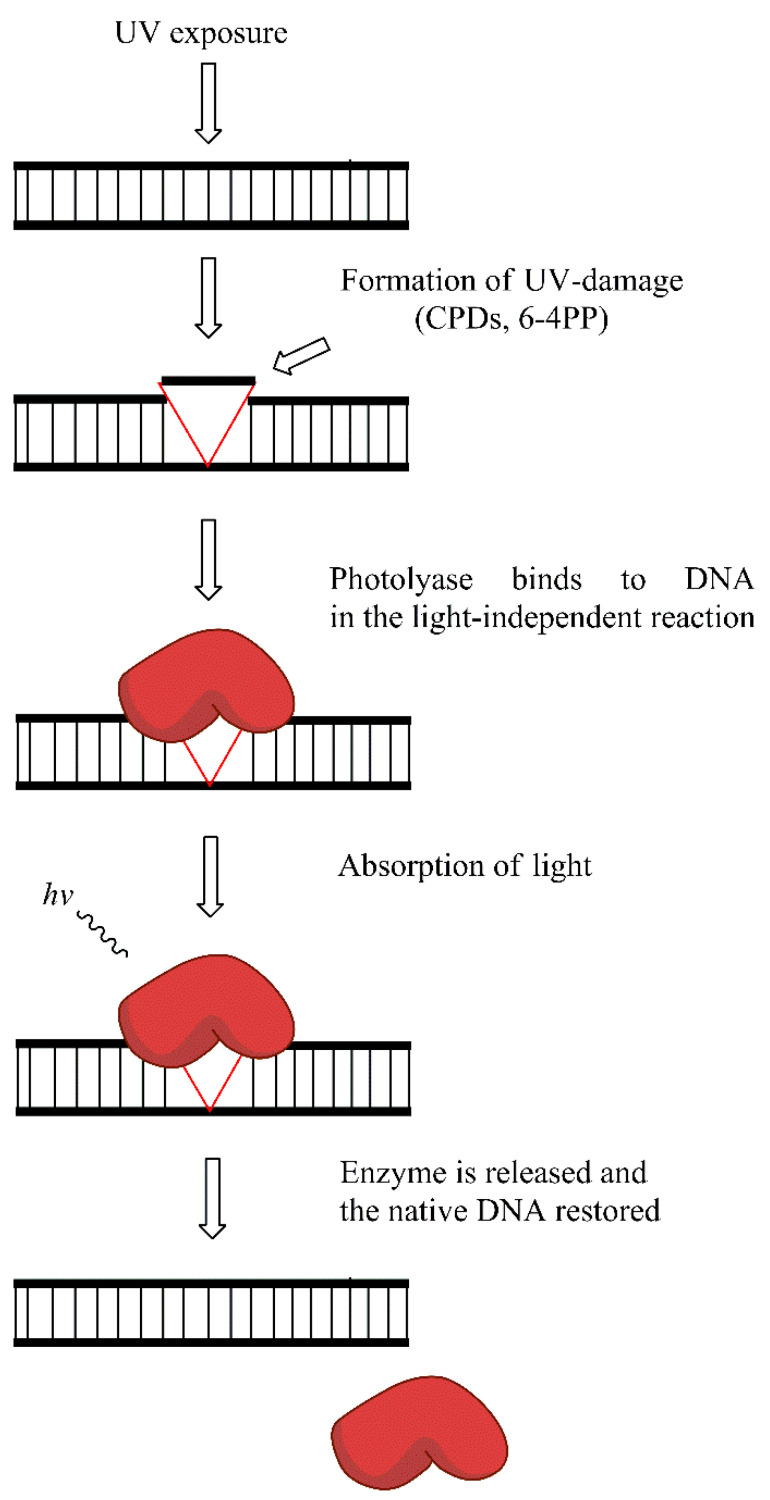
Photoreactivation reaction: the exposure to UV light leads to the formation of cyclobutane-pyrimidine dimers (CPDs) and 6-4 photoproducts (6-4PPs) removed by photolyase enzyme in the light-dependent manner.

Placental mammals (including humans) lack photolyase activity, and the repair of UV-induced damage is probably replaced by the activity of NER. However, in many organisms, photolyases are the key enzymes that remove photo-damage. CPDs and 6-4PPs are the two main types of photolesions repaired by photolyases. In the absence of blue light photolyases bind to CPDs, and 6-4PPs. The absorption of light by the class specific chromophore (either 5,10-methyl tetrahydrofolate (MTHF) or 8-hydroxy-5-deazaflavin (8-HDF)) leads to its excitation. The energy obtained through excitation of chormophore is then transferred to important cofactor—reduced flavin adenine dinucleotide (FADH). Consequently, photoexcitated FADH provides energy for the dimer-splitting. Subsequent back electron transfer regenerates the FADH, ensuring the proper FAD form for the next reaction. A thymine pair is consequently flipped back into the DNA duplex and the hydrogen bond between thymine and adenine is formed. Two classes of CPD photolyases with different pathway of electron transfer, and different mechanistic properties exist. The repair of 6-4PPs is different than CPDs and requires (6-4PPs) photolyase and involves oxetane intermediate formation. However, conflicting results exist on the oxetane formation during repair. Again, the covalent links between adjacent pyrimidines in the dimers are broken, and the proper structure of DNA molecule is restored [77,78,79,80,81]. The simplistic model of photoreactivation is presented in Figure 7.

You et al. [82] showed that CPDs are accountable for nearly 80% of the UV-B-induced mutations in mouse cell lines equipped with either CPD or (6-4PP) photolyase. However, photoreactivation with (6-4PP) photolyase did not lower the mutation frequency significantly in reporter genes [82]. This is concurrent with studies of another research group. Schul et al. [83] created two mouse lines carrying either two or three copies of CPD photolyase transgene from *P. tridactylis*. The insertion of the transcript has not affected mice physiology in terms of viability, development, and the ability to reproduce. The team investigated CPDs specific repair upon irradiation with UV-C light of fibroblasts expressing photolyase followed by subsequent exposition of cells to photoreactivating light (repair-inducing light). The results obtained from cell culture studies confirmed enzymatic activity of marsupial photolyase, and enhanced removal of CPDs in mouse cells. Consequently, the removal of CPDs in the skin of transgenic mice was assessed in vivo. Again, after exposure to photoreactivating light, the enhanced removal of CPDs was observed with concomitant decrease in apoptotic response of skin cells exposed to UV light. Additionally, the team obtained similar results in basal keratinocytes of transgenic mouse line (K14-CPD-1) containing approximately 25 copies of the photolyase transgene, except that the lesions were effectively removed in the basal region of the epidermis, not in the dermis, and the upper layers of the epidermis. This indicate that the photoreactivation of CPDs may be employed for the certain compartment of the skin. In conclusion the team developed transgenic mouse lines that express the CPD photolyase in all tissues (employing the β-actin promoter), and with selective expression of the repair protein in the basal region of the epidermis. Moreover, the expression of photolyase significantly enhanced the removal of CPDs, and prevented cell apoptosis upon DNA damage [83]. Later, Jans et al. [84] generated mice expressing the *A. thaliana* (6-4PP) photolyase from the ubiquitous β-actin promoter, and both CPD and (6-4PP) photolyase expressing transgenic mice. Consequently, researchers obtained similar results to previous research. The incorporation of (6-4PP) photolysase increased the removal of photolesion in mouse fibroblast. However, without enhanced removal of CPDs. Furthermore, breeding of (6-4PP) photolysase expressing mice with CPD-photolyase resulted in generation of double-transgenic animals with enhanced removal of both CPDs, and 6-4PP after exposure of cells to photoreactivating light. However, photoreactivation of 6-4PPs did not significantly enhanced cellular survival determined by 3H-thymidine incorporation survival assay, indicating that CPD-photolyase activity is the major factor influencing cell survival upon UV damage. Furthermore, CPD-photolyase/6-4PP-photolyase mice fibroblasts exhibit a UV sensitivity similar to that observed in CPD-photolyase cells. Taken together all these findings, and subsequent evidence that CPDs largely influence the RNA synthesis or cell death, this type of photolesions is thought to be the major contributor to deleterious consequences of UV exposure. Furthermore, *A. thaliana* (6-4PP) photolyase is active in skin cells of mouse and contributes to the efficient repair of 6-4PPs after exposure to photoreactivating light without a decrease in apoptotic response. In the case of double transgenic mice, the decline in apoptotic response is similar to that observed in mice expressing only CPD-photolyase. Importantly, researchers examined the contribution of CPDs, and 6-4PPs to mutagenic events. The results indicate that CPDs are the major lesions that contribute to mutagenic events, and UV-induced carcinogenesis compared with 6-4PP [84].

### 8.2. UVDE-mediated Repair (UVER)

In some organisms such as bacteria and yeast additional excision repair (besides base excision repair (BER) and NER) exist. The reaction is carried out by an enzyme called UV damage endonuclease (UVDE). UVDE introduces nicks immediately 5′ to specific type of lesions (CPDs, 6′-4′PP, base damage, and others) and initiates UVDE-mediated excision repair (UVER or UVDR). The repair is rapid and functions properly even in the absence of NER. UVDE introduces a nick on the 5′ end of the photoproducts, leaving a 5′-P-UV lesion and a 3′-OH end. The free 3′-hydroxyl group is a substrate for excision repair strand displacement DNA synthesis. Studies in *S. pombe* revealed the rad2 gene as an important factor for UVDE-mediated repair. The gene encodes a structure-specific flap endonuclease that is involved in processing of UV damage into single-strand breaks (SSBs). UVER may play a crucial role in repair of photoproducts as it removes CPDs more rapidly than NER, especially on the transcribed strand. Moreover, 6′-4′PP are removed more efficiently than CPDs suggesting important role of UVER in the damage removal. The role of UVER as a repair pathway should not be neglected. However, caution should be taken when overestimating the role of UVDE in repair processes, as NER still remains the most efficient damage removal system. The major disadvantage of UVER is certainly the great number of nicks introduced in the DNA strand that may comprise an obstacle for replication process [85,86,87].

### 8.3. Nucleotide Excision Repair (NER)

Nucleotide excision repair (NER) is the main UV-damage removal pathway both in prokaryotes and eukaryotes. Humans possess only NER mechanism for the repair of dipyrimidine DNA lesions. Furthermore, NER removes a wide variety of damage such as bulky DNA adducts, ROS-damaged bases, and damage resulting from crosslinking agents. The common feature of the aforementioned damage is certainly the strong perturbation of the DNA helix [88,89]. Prokaryotic NER is far poorer in terms of protein complexes involved in repair. However, in both taxa, NER can be divided into several common steps: (a) damage detection, (b) damage verification, (c) incision, (d) excision, and (e) repair synthesis and ligation. Table 1 presents the main NER components in both prokaryotes and eukaryotes.

In prokaryotes, DNA damage is detected by dimer of UvrABC system protein (UvrA) working together with UvrB. The detection step can also be carried out by transcriptional-repair coupling factor (TRCF, also known as Mfd). The transcription-coupled repair is induced mainly by the stalling of RNA polymerase at the sites of damage. TRCF allows for the removal of RNA polymerase from the damaged site and subsequent UvrABC-mediated repair. The UvrA dimer is the key complex for the damage recognition that scans DNA in the search of damage, while UvrB catalyzes the local dissociation of the DNA helix and provides a scaffold for UvrC protein. In the next step, the UvrA dimer dissociates form the complex, leaving UvrB bound to the DNA waiting for UvrC engagement in the repair process. UvrC acts as an endonuclease that nicks the DNA 7-8 nucleotides from 5′-end and 4–5 nucleotides from the 3′-end of the damaged site. Alternatively, UvrC homolog excinuclease (Cho) may be incorporated in the DNA process; however, this nuclease cleaves DNA molecule 4 nucleotides farther away than the normal cleavage site on the 3′ side of the lesion. The 3′-incision precedes the 5′-incision for both Cho and UvrC-mediated repair. Finally, 12–13 nt. long damage containing fragment is released (16 nt in the case of Cho protein). In this process, the UvrD protein acts as a helicase that removes hydrogen bonds between the complementary nucleotides. The DNA polymerase I (Pol 1) and DNA ligase restores the proper DNA sequence [90,91,92]. 

Eukaryotic NER can be divided into global genomic NER (GG-NER) and transcription-coupled NER (TC-NER) [89]. The first step both for GG-NER and TC-NER is the chromatin remodeling process, which allows a multitude NER proteins access to the sites of damage [93,94]. In GG-NER, damage is recognized by the XPC protein working in concert with the UV excision repair protein RAD23 homolog B (RAD23B) and centrin 2 (CETN2) [23]. The main function of the formed complex is DNA scanning in search of helix destabilization. XPC binds to the undamaged strand opposite to the photodamage [95]. CPDs that comprise to smaller disturbances in the DNA helix are detected by UV-DDB complex consisting of DDB1 (XPE-binding protein) and specific for GG-NER—DDB2 protein. The complex binds to the UV damage and stimulates the incorporation of XPC protein in the repair process. Substrate-bound XPC becomes a substrate for transcription factor II (TFIIH) complex encompassing 10 protein subunits, including XPB (3′-5′ helicase) and XPD (5′-3′ helicase) ATP-dependent helicases. The interaction between TFIIH and XPC-RAD23B allows (through XPB) binding of XPD and subsequent complex move on the DNA strand. XPD fixed at the damage site recruits XPA, RPA, and XPG, leading to the formation of a pre-incision complex. The RPA protein coats ssDNA resulting from the action of XPB and XPD and forms a platform for XPG and ERCC1-XPF endonucleases. The first incision is introduced in the 5′end of the existing damage by ERCC1-XPF, while the second is introduced by XPG on the opposite side. As a result, a 25–32-nt.-long DNA fragment containing damage is released. In the next step, DNA polymerase δ/ε works with important cofactors (proliferating cell nuclear antigen (PCNA) and replication factor C (RFC)) to resynthesize the lost DNA fragment. Finally, DNA ligase I or ligase IIIα, working together with XRCC1, ligates the broken phosphodiester bonds and the chromatin status form before the repair is restored [95,96].

TC-NER is the repair closely connected with actively transcribed genes. This is of particular importance, given the fact that transcriptional arrest following DNA damage interfere with gene expression influencing cell growth and survival. In contrast to other generally mutagenic repair mechanisms such as translesion synthesis (TLS), TC-NER resolves lesions with high rapidness and fidelity. This prevents formation of mutant transcripts and is pivotal for the cellular homeostasis. Moreover, blocked transcription complexes are extremely cytotoxic structures. Thus, TC-NER protects the cells from apoptosis and cell cycle arrest in case of robust DNA damage response. TC-NER is initiated in response to stalling of RNA polymerase II (RNAPII) at the sites of damage. Stalling of RNAPII at sites of UV damage immediately activates TC-NER. The repair begins with the recruitment of CSA(ERCC8) and CSB(ERCC6) to the lesions [97]. CSA together with DDB1, Cullin 4A and E3 ubiquitin-protein ligase RBX1 (RBX) proteins works as a E3-ubiquitin ligase. However, upon damage occurrence the ligase is inactivated by constitutive photomorphogenesis 9 (COP9) signalosome (CSN) that deneddylates Cullin 4A. CSA is also crucial for recruitment of other TC-NER components described further below. On the other hand, CSB protein is a DNA-dependent ATPase and nucleosome remodeling enzyme involved in transcriptional control of genes following UV irradiation. Upon binding with RNAPII, CSB alters the DNA conformation by wrapping the DNA around the protein itself and changing the association of RNAPII with DNA. It may also stimulate the assembly of other components of TC-NER complex [98] Binding of CSA-CSB complexes leads to reverse translocation of RNA polymerase from the damage sites and expose the damage for repair proteins including XPA-binding protein 2 (XAB2), ubiquitin-specific-processing protease 7 (USP7) and high mobility group nucleosome-binding domain-containing protein 1 (HMGN1) [99]. Down-regulation or mutation in the former factors results in impaired repair of lesions in transcribed strands and lead to apoptosis of cells harboring damage [97]. XAB2 interacts with XPA protein and RNAPII, playing role as a scaffold for other TC-NER components. HMGN1 also interacts with RNAPII in a CSA/CSB dependent manner and works as a nucleosome binding protein that competes with histone H1 for binding with chromatin in linker regions [99]. USP7 works as a ubiquitin carboxyl-terminal hydrolase and possess multiple roles in the DDR. Furthermore, it interacts with another protein UV-stimulated scaffold protein A (UVSSA) and this interaction is critical for restoration of gene expression upon UV irradiation [100]. The detailed information on this topic can be found in the following references [100,101,102,103,104]. Moreover, backtracking of RNAPII allows correction of misincorporated nucleotides in RNA:DNA hybrids within transcription bubbles via cleavage of 3′ protruding RNA parts with mismatched nucleotides. This event may be promoted by transcription factor TFIIS that acts as a stimulator of RNAPIIs mRNA-cleavage activity. The rest of the pathway is quite analogous to GG-NER [95,96,105]. Repair of damage both in GG-NER and TC-NER requires chromatin remodeling for the enhanced access of pathway factors to existing damage. Moreover the NER pathway in eukaryotes is tightly regulated by post-translational modifications and chromatin dynamics reviewed in [89,106,107,108,109]. 

### 8.4. Base Excision Repair (BER)

Base excision repair is highly conservative pathway responsible for the repair of damage that arise during alkylation, deamination, and oxidation of DNA bases. Both prokaryotes and eukaryotes utilize homologous proteins with similar function for efficient repair. However, eukaryotes possess several additional enzymes [110]. The repair both in prokaryotes and eukaryotes is initiated by DNA glycosylases that scan, and search for small distortions in DNA helix then hydrolyze the N-glycosidic bond of the damaged base leaving an AP-site. There are two main classes of glycosylases—monofunctional, and bifunctional [111].

Monofunctional glycosylases possess only glycosylase activity. Bifunctional glycosylases possess additional AP lyase activity that results in formation of 3’ α,β-unsaturated aldehyde, and 5’ phosphate (β-elimination mechanism) or 3′, and 5’ phosphates (β/δ-elimination mechanism) after cleavage of AP-site. AP-sites that arise during monofunctional glycosylase activity are further processed by AP-endonulceases (APE) that cleave the AP-site leaving 3’ hydroxyl, and 5’ deoxyribosephosphate (5′dRP) ends. Number of end processing enzymes that allow formation of ligatable DNA ends exist. These include PNKP that removes 3′phosphates leaving 3′OH group, and phosphorylates 5’ hydroxyl ends of DNA molecules, aprataxin (APTX) that removes adenosine monophosphate (AMP) from DNA ends after abortive activity of DNA ligase IV during NHEJ, and tyrosyl-DNA phosphodiesterase 1 (TDP-1) that remove covalent adducts from DNA through hydrolysis of a 3’-phosphodiester bond. After the excision of damaged base poly (ADP-ribose) polymerase-1 (PARP-1) is recruited to the sites of damage to ensure high efficiency of the repair process. APE1 process 3’ α,β-unsaturated aldehydes into 3′OH ends ready for ligation. In human, Polβ is the main polymerase engaged in short patch BER. Moreover, it possesses lyase activity that removes 5’ 5′dRP formed during AP-cleavage by APE. The ligation process is carried by DNA ligase III working together with its cofactor X-ray repair cross-complementing protein 1(XRCC1) [111,112,113]. This repair process is known as a short patch BER. 

In contrast to short-patch BER, where one nucleotide is introduced during repair process, long-patch BER involves the resynthesis of longer (up to 13 nucleotides) fragment of DNA molecule. The synthesis is carried by high-processivity polymerases (pol δ, and pol ε) working together with cofactors such as PCNA, and PCNA loading factor RFC. Encounter of DNA polymerase with DNA damage results in 5′flap formation. The resulting flap is cleaved by another end processing enzyme flap endonuclease (FEN1). Finally, DNA ligase I ligates the break in long-patch BER [25,113,114].

**Figure 8:**
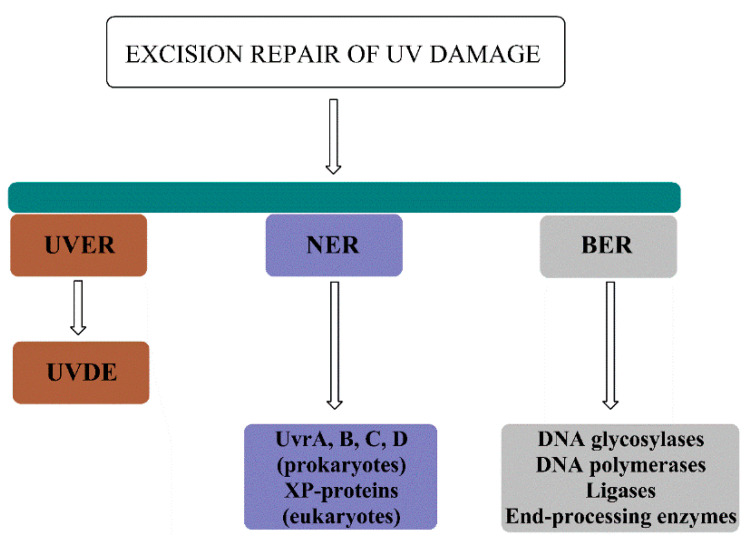
Excision repair pathways for the repair of UV damage include UV-damage endonuclease (UVDE)–mediated repair (UVER), nucleotide excision repair (NER) composed of Uvr proteins in prokaryotes and XP proteins in eukaryotes, and base excision repair (BER) for the repair of UV-induced oxidative damage.

Together, UVER, NER and BER comprise the pool of excision repair pathways utilized to repair the UV damage (Figure 8).

### 8.5. Double Strand Break Repair (DSBR)

Double-strand breaks (DSBs) are a group of lesions commonly observed following UV-B irradiation of cells. However, UV radiation does not directly induce DSBs. Both photolesions (CPDs and 6-4PPs) and ROS may lead to the formation of DSB and this is often associated with transcription or replication blockage. Furthermore, DSBs may arise during repair processes such as BER pathway. Although DSBs do not occur frequently, they constitute a group of the deadliest forms of DNA damage that cell may encounter during a lifetime. Repair of DSBs is quite difficult. To ensure that such a detrimental damage will be repaired, cells evolved complex ATM and ATR signaling events leading to activation of the two basic DSB repair mechanisms compared in Table 2 [115,116,117].

One of the basic mechanisms, called homologous recombination (HR), is based as the name suggest on the homology and uses sister-chromatid sequences as a template. HR is therefore restricted to S and G2 phase of cell cycle where condensation status of the chromatin allows homology searching. HR can generally be divided into four steps: 5′-3′ resection, strand invasion on the homologous strand, repair synthesis and strand separation. Homology searching requires fragments of single stranded DNA (ssDNA). These fragments are generated by the activity of MRN complexes. Double-strand break repair protein (MRE11), one of the components of MRN possess endo and exonucleolytic activity that enables single strand DNA (ssDNA) formation [55,118]. Resection step is controlled by CtBP-interacting protein (CtlP), which interacts with BRCA1-C. Other nucleolytic enzymes may also take part in resection phase of HR. These include exonuclease 1 (EXO1), DNA replication ATP-dependent helicase/nuclease DNA2 (DNA2), Bloom syndrome protein (BLM), and Werner syndrome ATP-dependent helicase (WRN). ssDNA is then coated with RPA protein that comprise to the stability of DNA fragments and form a platform for DNA repair protein RAD51 (RAD51) protein [55]. RAD51 is another key component of the HR repair machinery involved in nucleofilament formation and homology searching during HR. In this step, RAD51 works together with BRCA2 to allow the strand invasion onto undamaged DNA duplex [119,120]. 

**Figure 9:**
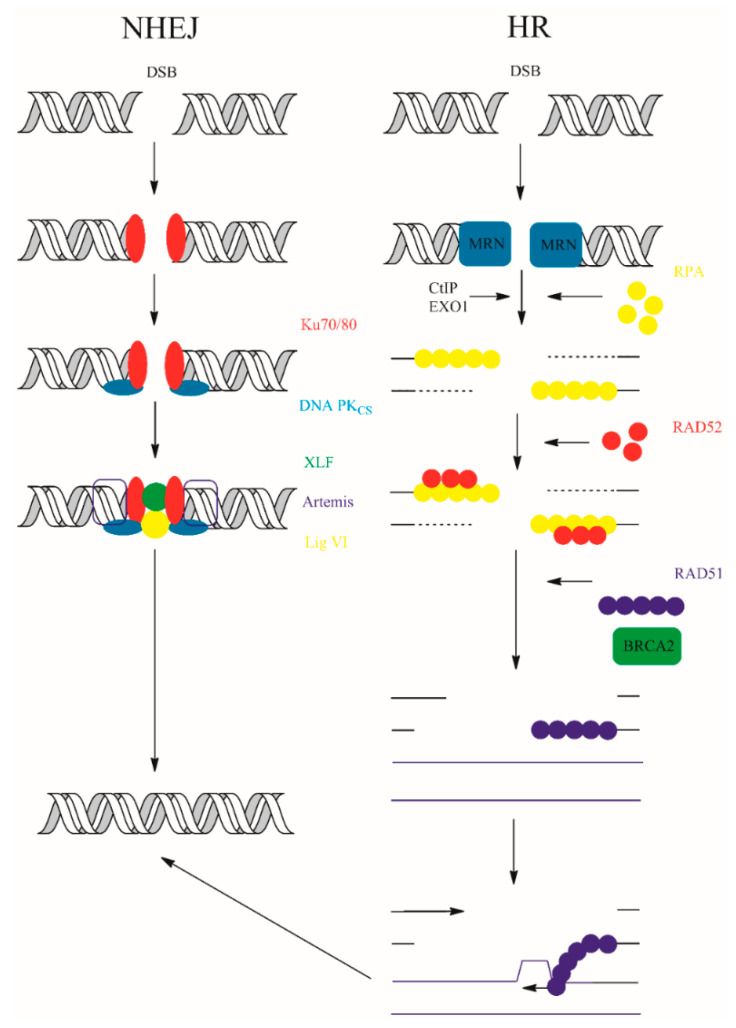
NHEJ and HR double strand break repair. The details of the repair processes were placed in the main text above.

In contrast, NHEJ is active throughout the cell cycle. The sensory X-ray repair cross-complementing protein 5/6 (Ku70/80) and DNA-PKcs form synapses, a DNA-protein complexes with broken DNA ends [121]. Many other components: X-ray repair cross-complementing protein 4 (XRCC4), XLF (Cernunnos), or DNA ligase IV participate together with synapse components for efficient repair. Non-ligatable ends can be connected following processing with accessory enzymes ARTEMIS, aprataxin (APTX), and PNK-like factor (APLF). Two variants of canonical NHEJ pathway exist—Ku-independent NHEJ pathway microhomology-mediated end-joining (MMEJ) and alternative end-joining (AEJ)—but their activity is connected with sequence deletions [4]. The participation of DNA polymerase in the NHEJ pathways is still questionable. However, some studies suggest participation of DNA polymerase λ (Pol. λ) and/or polymerase μ (Pol. μ) in repair process [122,123,124]. The schematic representation of eukaryotic NHEJ and HR is shown in Figure 9.

### 8.6. Translesion Synthesis (TLS)

Cells replicate the genetic material with high fidelity. The main replicating, highly processive eukaryotic DNA polymerases Pol δ and Pol ε ensure the genomic stability and prevent mutagenesis. Moreover, replicating polymerases are essential constituents of repair pathways. A wide repertoire of polymerases exists in cells. These polymerases vary in the fidelity and processivity. Damage tolerance is certainly an unusual repair mechanism employed in response to UV radiation. It allows rapid prevention of consequences of the replication fork arrest [125]. Furthermore, it provides additional time for the repair (until released in response to damage DNA polymerase returns to finish the replication process). In contrast to classical repair pathways TLS synthesis do not restore proper sequence and structure of DNA molecule. Characteristic feature of TLS polymerases is certainly their mutagenic potential, due to low fidelity and the fact that the lesion is still present in the DNA molecule. In eukaryotic cells Y family (Polη, Polι, Polκ), B family (Polζ), X family (hPol β, λ, μ), and A (θ oraz ν) family of polymerases are implicated in repair processes [126]. Additionally, Rev1 highly specialized dCMP transferase also exist [125].

The active sites of TLS polymerases are more spacious and accommodate a variety of non-canonical Watson-Crick base pairs compared to replication polymerases. These active sites may be so bulky that are able to accommodate two covalently linked bases in pyrimidine dimers. However, this ability significantly reduces the fidelity of the enzymes. Moreover, TLS polymerases lack the 3′-5′ exonucleases (proofreading) activity further limiting repair accuracy [125,127]. The insights on TLS came from studies in bacteria and yeast. The TLS activity was shown in UV-induced phage reactivation experiments. Later, Rodman identified the bacterial SOS model for error-prone repair mechanisms of UV damage. Three TLS polymerases were shown to be regulated by pivotal SOS regulator/repressor LexA: Pol II (polB), Pol IV (dinB) and Pol V [128].

In the repair of photoproducts, Pol III incorporates nucleotide opposite to the first T of a T-T located on the 3′end of the CPD. This requires the recombinase (RecA) recruitment to the DNA near the lesion. Interaction between Pol III and UmuDC (Pol V) allows incorporation of second T on the 5′end of the CPD. One of the incorporated bases is not the classical Watson–Crick base pair and thus may lead to mutagenic events. Moreover, polymerase switching may occur in the repair process. The simplest example of the coordinated action of polymerases in repair process is *E. coli* Pol IV and Pol II switching with Pol III on the β-sliding clamp at a stalled replication fork [125].

Eukaryotic TLS polymerases also have the ability to synthesize over UV-DNA damage. Pol η and Pol ζ can bypass T-T CPDs and were shown to play an important role in TLS of UV damage, while REV1 was shown to be required for bypass of a 6-4 TT dimer in vivo. Particularly, Pol η is crucial for UV-damage bypass synthesis as it allows continuous DNA synthesis past sites of UV damage and contributes to stalled replication forks restart. Furthermore, it prevents the formation of double strand breaks in response to UV radiation [125,127].

The TLS activity is accurately controlled in order to ensure the repair to occur where and when is needed. In eukaryotic cells interaction of polymerases with PCNA (dependent on its ubiquitination status) seems to be crucial for employment of TLS polymerases for lesion bypass [127,129]. However, conflicting results on the monoubiquitination of the PCNA and TLS synthesis exist [130]. Additionally, TP53 and P21 may act to suppress TLS activity and stimulate UV-induced ubiquitination of the PCNA. Moreover, REV1 seems to regulate TLS via recruitment and coordination of other proteins involved in the damage response [127]. Excellent reviews on TLS are available and thus we refer the reader to reference list [125,127,131,132,133,134,135,136,137,138,139].

### 8.7. DNA Repair in Mitochondria

Mitochondria are especially susceptible to oxidative damage due to several factors such as a) lack of protective histones, b) lack of introns, c) direct proximity of mtDNA and electron transport chain where ROS may be generated, and d) less efficient repair of emerging damage. Exposure to UV light induces low levels of ROS that contribute to DNA damage. However, UVR is responsible for the generation of a higher amount (approximately 10-fold increase) of mutations in mtDNA than in nuclear DNA. Due to the lack of introns, mutagenesis is more likely to affect a coding region compared to nuclear DNA. Therefore, the arising mutations in genes encoding proteins engaged in electron transfer rise the possibility of synthesis of defective respiratory chain proteins and may lead to exacerbation of the damage process via production of excessive amount of ROS. This is known as a “mitochondrial vicious cycle” [140,141]. mtDNA do not encode repair machinery. Therefore, all the repair proteins are transported to mitochondria after exposure to damaging agents. BER is the main repair pathway present in mitochondria. However, the repair synthesis in mtBER is carried out by POLG, the main mitochondrial polymerase and the ligation is performed by ligase III. Furthermore, SSBs and DSBs generated as products of replication stalling, ROS activity and abortive topoisomerase action may be repaired by either HR or NHEJ [142,143].

Exposure of cells to UV-C light induces the formation of CPDs and 6-4PPs in mtDNA. Furthermore, 6-4PPs are produced in higher yields in mtDNA compared to nuclear DNA. Surprisingly, mitochondria do not exhibit NER activity and thus the removal of photoproducts can be significantly reduced [141,144]. However, studies performed on *S.pombe* revealed that certain photoproducts such as CPDs and 6-4PPs can be repaired via UVDE-mediated repair in mitochondria, thus leaving mtDNA without an alternative to repair UV damage. Additionally TLS synthesis over the photodamage was also detected in mitochondria [145].

## 9. Disease Relevance and Future Therapeutic Strategies

Defects in DNA damage response are the underlying cause of multitude human disease [52,146,147,148]. UV radiation causes variable reactions in human cells. The versatility of effects implicates different human diseases related to UV damage and determines the susceptibility of the individual to sunlight. The most prevalent group of diseases directly related to higher vulnerability to UV light comprise genetic disorders with defects in DNA repair mechanisms. These include the conditions with mutations in NER pathways (presented in Table 3) such as xeroderma pigmentosum (XP) [149,150], Cockayne syndrome (CS) [151,152], trichothiodystrophy (TTD) [153,154,155,156], UV-sensitive syndrome (UV-SS), and cerebro-oculo-facioskeletal syndrome (COFS) [157,158,159]. Different mutations of a given gene influence the clinical outcome. This is a result of variable yields and structures of the mutated gene products. Moreover, NER may be involved in other metabolic processes and this may contribute to variance in clinical features. XP is a relatively rare autosomal recessive genetic disorder with extreme sensitivity to sunlight. Hypopigmentation and hyperpigmentation are two main cutaneous features of XP patient. Moreover, XP patients with defective *XPC* or *XPE* genes have >1000 times higher incidence of cancer (10-fold the risk for visceral cancers). Moreover, other disease variants may be caused by mutations in *XPA*, *XPB/ERCC3*, *XPG*, *XPF*, and *XPV*. The observed higher incidence is attributed to the accumulation of DNA lesions such as CPDs, 6-4PPs, and cyclopurines that normally would be repaired by NER. Cockayne syndrome predominantly impacts tissues composed of non-proliferating or slowly proliferating cells, thus the main features include neurological symptoms and growth defects such as dwarfing, microcephaly, mental retardation, dysmyelination, and retinal degeneration. Patient cells defective in TC-NER include hypersensitivity to sunlight and various mutagens. The molecular basis of the disease includes mutations in *XPG*, *XPB*, *XPD*, *CSA* (*CKNI, ERCC8*), and *CSB* (*CKN2*, *ERCC6*) genes. *CSA* gene is mutated in 10–20% of cases, while *CSB* in 80–90%. TTD is another disease associated with mutations in NER genes. The alteration in one of the genes coding for TFIIH subunits—ERCC/XPD or ERCC3/XPB—leads to enhanced sensitivity to UV radiation, fragile hair and nails, and scaly skin. *TTDA* (general transcription factor IIH subunit 5) and *TTDN1* (trichothiodystrophy non-photosensitive 1) genes may also harbor a mutation; however, they do not contribute to UV sensitivity [107,160,161,162].

UV-sensitive syndrome (UV-SS) represents the mildest form of disease associated TC-NER. Patients with UV-SS exhibit sun sensitivity, however, do not display neurological defects and susceptibility to cancer. UV-SS is caused by inactivating mutations in the *UVSSA* gene encoding UV-stimulated scaffold protein A. However, mutations in other genes such as *CSA* and *CSB* may also be underlying causes of the disease. COFS is a rare, lethal, prenatal or neonatal neurodegenerative syndrome resulting from mutations in *CSB* gene but also *XPD* or *XPG* [107].

The traditional method of treatment of these diseases is certainly avoidance of exposure to sunlight. This, however, may be difficult for both patients and their families. The promising future methods of treatment include the use of gene therapy and topically administered creams containing repair enzymes latter discussed in this review [163,164].

Overexposure to sunlight is directly connected the with onset of skin cancers such as basal cell carcinoma (BSC) [165], squamous cell carcinoma (SCC) [166,167], and malignant melanoma (MM) [168,169]. The accumulation of unrepaired photolesions in the DNA and formation of ROS may cause mutations in proto-oncogenes or tumor suppressors (for example *TP53*) and lead to malignant transformation of cells. UV mutagenesis involves T → C transitions, the tandem CC → TT mutations and less prevalent mutations in CT-sites. In contrast, TT 6-4PPs are mutagenic because of T → C transitions. However, the repair capacity is the main determinant of how long the damage will persist in the DNA and thus how long it will contribute to mutagenic potential [15].

As the genome is the main target of the UV damage, it is reasonable that the protection of the DNA by other UV-absorbing molecules may be the easiest and most achievable way to prevent cells from acquiring lesions. Normally, the skin is protected by natural pigments such as melanin, and its content is directly connected to the formation of photolesions. The higher the melanin concentration, the lower yields of CPDs generated in the DNA of exposed skin cells [170]. Antioxidant enzymes such as catalase (CAT), glutathione peroxidase (GPX), glutathione reductase (GR), superoxide dismutase (SOD), and thioredoxin reductase (TRX) represent major radical scavenging defense against ROS [171]. Moreover, the use of natural or synthetic antioxidants as supplementary components of artificial sunscreens may enhance the protective effects of mineral (e.g., titanium dioxide (TiO2) and zinc oxide (ZnO)) and organic UV filters. The detailed classification of sunscreen agents has been summarized elsewhere [172]. Plants are the major source of natural antioxidants used in sun protective formulations due to the high concentration of polyphenols, monoterpenes, flavonoids, organosulfides, and indoles [173]. However, their use can be limited due to photo-fragmentation and/or photoisomerization reactions, energy and electron transfer reactions that may lead to undesirable reactions between formulation components and result in the generation of reactive byproducts. Thus, the safety of these sunscreens need to be elucidated [174]. Moreover, there is a need for more standardized and unified testing methods for comparison of the effects of UV radiation on skin, as they are usually based on erythema formation, and this may not fully reflect the protective roles of sunscreens [175].

Moreover, UVR also has local and systemic immunosuppressive properties that is manifested by susceptibility to viral or bacterial infections and increased predisposition to cancer as an effect of compromised surveillance against tumors. Thus, it is crucial to understand the immunological response to UV radiation in the light of constantly increasing exposure to sunlight due to the depletion of the ozone layer [170]. Moreover, bacteria, like other organisms, utilize various repair pathways to counteract genomic threats, including those used to exterminate them. As a consequence, drug resistance is emerging. Understanding the repair pathways may lead to the discovery of new molecular targets for novel antibacterial drugs [110]. Moreover, UV-C could be used as germicidal agent applied directly to the infection sites of the skin and employed to kill bacteria and enhance wound healing. The most important advantage of controlled UV radiation over antibiotic use is certainly the low cost and faster eradication time. Moreover, light has many more advantages over disinfectants, biocides, and anti-infectives, including (a) environmental friendliness, (b) nontoxicity, (c) rapidness, and (d) no microbial resistance to UV light. The wavelength, exposure time, and the radiation source are the main factors that will contribute to the applicability of the radiation in the clinic. As UV light may provide both advantages (in the form of facilitated healing) and disadvantages (DNA damage), all the benefits and risks must be taken into account [176,177,178,179]. Furthermore, understanding of the consequences of sunlight exposure have significant implications for biothreat control and bioterrorism. The knowledge regarding response of different microorganisms to UV light may help control the spread of various pathogens including viruses, bacteria, fungi, and parasites [179,180].

As previously mentioned, some attempts were carried to directly deliver the enzymes engaged in removal of UV lesions. Clinical trials with the use of topically applied liposomal formulation with bacteriophage T4 CPD damage removal enzyme – T4 endonuclease V resulted in lowered actinic keratoses and basal-cell carcinoma incidence in XP-patients [181,182]. Subsequently the same approach was undertaken with photolyase enzyme and suggested more effective removal of CPDs [183,184]. The outcomes of clinical research concerning the use of photolyase as ”sunscreens” and the comparison of use of both T4 endonuclease V and photolyase enzymes in protective creams have been summarized elsewhere [184,185]. However, several questions remain unanswered, as were suggested by Yarosh et al.: (a) Do DNA repair enzymes really work? (b) Do the enzymes penetrate the skin and repair DNA? (c) What are the damage repair capacities of these enzymes? (d) Does the repair significantly lower skin cancer incidence? (e) Do the enzymes have only preventative role or are able to repair past damage? (f) How do DNA repair enzymes influence skin photoaging [186]?

## 10. Conclusions

Cells are constantly exposed to various environmental factors that damage genomic DNA and pose a threat for their proper function. DNA is the main target of UV radiation that causes the formation of many different types of damage, including the most prevalent photoproducts, such as cyclobutane-pyrimidine dimers (CPDs) and 6-4 photoproducts (6-4PPs), DNA bases damaged by ROS, and strand breaks. To counteract such deleterious factor, cells have evolved DNA damage responses (DRRs) that prevent the consequences of damage. In lower organisms such as bacteria, three distinct repair systems remove the photodamage: photolyases remove CPDs and 6-4PPs in a light-dependent reaction, UVDE endonuclease cleave the DNA directly 5′ to the lesions, and the bacterial nucleotide excision repair (NER) pathway consisting of UvrA,B,C and D proteins work together in a multistep process to remove the existing damage. In eukaryotes CPDs and 6-4PPs are removed mainly by multi-protein NER pathway with an excised DNA fragment of 25-32 nt. long, that is further rebuilt in a repair synthesis step. On the other hand, damage caused by ROS induced in response to UV radiation as well as single strand breaks are repaired both in prokaryotes and eukaryotes via BER pathway. In contrast, DSBs are repaired via non-homologous end-joining (NHEJ) and homologous recombination (HR) pathways.

Genetic defects in the repair pathways (predominantly NER) lead to the onset of serious conditions such as xeroderma pigmentosum (XP) with extreme sensitivity to sunlight and predisposition to cancer. Moreover, excessive exposure to UV radiation is directly related to the formation of basal cell carcinoma (BSC), squamous cell carcinoma (SCC), and malignant melanomas (MMs). The knowledge regarding molecular basis of pathologies associated with impaired DNA damage repair may lead to better understanding of cellular pathways and crosstalks considering repair and give insight to the contribution level of individual repair proteins in molecular mechanisms employed in response to UV radiation. The most effective way to protect oneself from UV radiation is certainly the use of protective clothing. However, other protective measures have been proposed. These include the use of various sunscreen formulations. However, the potential benefits of sunscreen use need to be revisited, and photochemical interactions between individual formulation components seems crucial for the development of more effective sunlight screens. Moreover, topical delivery of repair enzymes (in the form of novel formulations) may help to alleviate the disease symptoms and prevent UV-induced cancers. However, many questions regarding its use remain to be answered.

## Figures and Tables

**Table 1:** Function of the nucleotide excision repair (NER) pathway components in bacteria (*Escherichia coli*) and *Homo sapiens*.

*E. coli* Protein	Function	*H. sapiens* Protein	Function
UvrA	Damage recognition, molecular matchmaker	DDB1, XPC, XPE, RAD23B	Damage binding, recruitment of other NER factors
UvrB	Damage recognition, unwinding and bending of DNA molecule	XPB XPD	3′-5′ helicase 5′-3′ helicase DNA unwinding
UvrC	3′ and 5′ excision	XPG XPF ERCC1	Endonuclease/3′ incision Endonuclease/5′ incision
UvrD	Helicase	XPA	Damage verification, RPA and ERCC1 recruitment
TRCF (Mfd)	Transcription coupled repair	CSA/CSB complex	Transcription coupled repair

**Table 2:** Comparison of eukaryotic homologous recombination (HR) and non-homologous end joining (NHEJ). Based on work by Jackson SP1 and Bartek J 2009 and Pennisi et al. 2015 [4,118].

Feature	Homologous Recombination (HR)	Non-Homologous End Joining (NHEJ)
Type of lesion repaired	DSBs, stalled replication forks, inter-strand DNA cross-links and DSBs resulting from abortive topoisomerase II action	Radiation- or chemically induced DSBs, V(D)J recombination
Key components	RAD51 and RAD51-related proteins (XRCC2, XRCC3), RAD52, BRCA2, RPA, FEN1, DNA polymerase and associated factors. Promoted by MRN, CtIP, BRCA1, and the ATM signaling pathway	Ku and DNA-PKcs, XRCC4, XLF/Cernunnos ligase IV. MRE11-RAD50-NBS1 complex, Artemis nuclease, PNK, Aprataxin and polymerases μ and λ
Mechanism	Repair involves invasion of the homologous strand on the DNA duplex	Repair involves formation of DNA-protein complexes with broken ends for efficient repair
Template	Requires sister chromatid as a template during repair process	Does not require sister chromatid as a template during repair process
Cell cycle	Is active only in S and G2 phase of the cell cycle	Is active throughout most of the cell cycle
Fidelity	Repair is generally error-free	Repair is error-prone

**Table 3:** Genetic disorders with NER deficiency.

Phenotype	Genes Mutated (Prevalent)	Genes Mutated (Others)	Features of Classical Variant of the Disease	Ref.
XP	*XPC*, *XPE*	*XPA*, *XPB/ERCC3*, *XPG*, *XPF*, *XPV*	extreme sensitivity to sunlight, hypopigmentation, hyperpigmentation, predisposition to cancer	[149,150]
CS	*CSA*, *CSB*	*XPG*, *XPB*, *XPD*	hypersensitivity to sunlight and various mutagens, neurological symptoms, dwarfing, microcephaly, mental retardation, dysmyelination, retinal degeneration	[151,152]
TTD	*XPB*, *XPD*	*TTDA*, *TTDN1*	sensitivity to sunlight, fragile hair and nails, and scaly skin	[153,154,155,156]
COFS	*CSB*	*XPD*, *XPG*	Depend on the subtype of the disease: neurodegeneration microcephaly, congenital cataracts, severe mental retardation, facial dysmorphism, and arthrogryposis	[107,157,158,159]
UV-SS	*UVSSA*	*CSA*, *CSB*	sun sensitivity	

## Abbreviations

53BP1	TP53-binding protein 1
6-4PPs	6-4 photoproducts
8-AIA	8-(5-aminoimidazol-4-yl)adenine
8-HDF	8-hydroxy-5-deazaflavin
AEJ	Alternative end-joining
AMD1	S-adenosylmethionine decarboxylase proenzyme
AMP	Adenosine monophosphate
ANP32E	Acidic leucine-rich nuclear phosphoprotein 32 family member E
ANP32E	Acidic leucine-rich nuclear phosphoprotein 32 family member E
AP1/2	Activating protein 1/2
APE	AP-endonulcease
aPKCs	Atypical protein kinase C
APLF	Aprataxin and PNK-like factor
APTX	Aprataxin
ATM	Ataxia telangiectasia mutated
ATR	Ataxia telangiectasia and Rad3-related protein
ATRIP	ATR-interacting protein
BAK	Bcl-2 homologous antagonist/killer
BAX	Apoptosis regulator BAX
BCL2	Apoptosis regulator BCL2
BER	Base excision repair
BIRC3	Baculoviral IAP repeat-containing protein 3
BLM	Bloom syndrome protein
BRCA1	Breast cancer type 1 susceptibility protein
BSC	Basal cell carcinoma
BTG1	BTG anti-proliferation factor 1
CAF-1	Chromatin assembly factor 1
CAT	Catalase
CCNG1	Cyclin G1
CDC25A/C	M-phase inducer phosphatases
CDKs	Cyclin dependent kinases
CDT1	DNA replication factor Cdt1
CENT2	Centrin 2
CHK1/2	Serine/threonine-protein kinase CHK1/2
Cho	Excinuclease Cho
COFS	Cerebro-oculo-facioskeletal syndrome
COP9	Constitutive photomorphogenesis 9
CPDs	Cyclobutane-pyrimidine dimers
CS	Cockayne syndrome
CSA/B	Cockayne syndrome protein A/B
CSN	Constitutive photomorphogenesis 9 (COP9) signalosome
CtlP	CtBP-interacting protein
DDB2	Damaged DNA-binding protein 2
DDR	DNA damage response
DDR1	Epithelial discoidin domain-containing receptor 1
DGPY	4,6-diamino-5-guanidinopyrimidine
DNA2	DNA replication ATP-dependent helicase/nuclease DNA2
DNA-PKcs	DNA-dependent protein kinase catalytic subunit
DP1	Transcription factor Dp-1
DR	Direct reversal of damage
dRP	5’ deoxyribosephosphate
DSBR	Double strand break repair
DSBs	Double strand breaks
DUSP5	Dual specificity protein phosphatase 5
EGFR	Epidermal growth factor receptor
ERKs	Extracellular signal-regulated kinases
EXO1	Exonuclease 1
FACT	Facilitates chromatin transcription
FADH	Reduced flavin adenine dinucleotide
FEN-1	Flap endonuclease 1
GADD45	Growth arrest and DNA damage-inducible protein GADD45 alpha
GG-NER	Global genomic NER
GPX	Glutathione peroxidase
GR	Glutathione reductase
HIRA	Histone regulator A
HMGN1	High mobility group nucleosome−binding domain−containing protein 1
HR	Homologous recombination
IER3	Radiation-inducible immediate-early gene IEX-1
INO80	Chromatin-remodeling ATPase
JNKs	C-Jun NH2-terminal kinases
KAP1	Nuclear corepressor KAP-1
KU70/80	X-ray repair cross-complementing protein 5/6
MAPK	Mitogen-activated protein kinase
MCM6	Minichromosome maintenance complex component 6
MCM7	DNA replication licensing factor 7
MDC1	Mediator of DNA damage checkpoint protein 1
MDM2	E3 ubiquitin-protein ligase MDM2
MM	Malignant melanoma
MMEJ	Microhomology-mediated end-joining
MRN	MRE11-RAD50-NBS1 complexes
mtDNA	Mitochondrial DNA
MTHF	5,10-methyl tetrahydrofolate
NAD+	Nicotinamide adenine dinucleotide
NBS1	Nibrin
NER	Nucleotide excision repair
NFAT	Nuclear factor of activated T cells
NFκB	Nuclear factor kappa B
NHEJ	Non-homologous end joining
NOLC1	Nucleolar and coiled-body phosphoprotein 1
ODC	Ornithine decarboxylase
P21	Cyclin-dependent kinase inhibitor 1A
PAR	Poly-ADP-ribose
PARG	Poly (ADP-ribose) glycohydrolase
PARPs	Poly [ADP-ribose] polymerases
PCNA	Proliferating cell nuclear antigen
PEA15	Astrocytic Phosphoprotein PEA-15
PI3K	Phosphoinositide 3-kinase
Pol	Polymerase
POLE3	DNA polymerase epsilon subunit 3
pRB	Retinoblastoma-associated protein
PTMA	Prothymosin alpha
PUMA	Bcl-2-binding component 3
RBX	Ubiquitin-protein ligase RBX1
RAD23B	UV excision repair protein RAD23 homolog B
RAD51	DNA repair protein RAD51
RAD9-RAD1-HUS1	Cell cycle checkpoint control protein complex
RARRES	Retinoic acid receptor responder 1
RecA	Recombinase A
RFC	Replication factor C
RNAPII	RNA polymerase II
RNF8/20/40/168	E3 ubiquitin-protein ligases RNF8/20/40/168
ROS	Reactive oxygen species
RPA	Replication protein A
RUNX3	RUNX family transcription factor 3
S100A11	S100 calcium binding protein A11
SCC	Squamous cell carcinoma
SOD	Superoxide dismutase
SRM	Spermidine synthase
SSBR	Single strand break repair
SSBs	Single strand breaks
ssDNA	Single strand DNA
STATs	Signal transducers and activators of transcription
SWI/SNF	Chromatin remodeling complexes
TAX1BP1	Tax1-binding protein 1
TC-NER	Transcription-coupled NER
TDP-1	Tyrosyl-DNA phosphodiesterase 1
TF	Transcription factor
TFIIH	Transcription factor II
TLS	Translesion synthesis
TNF-α	Tumor necrosis factor α
TOB1	Transducer of erbB-2 1
TOPBP1	DNA topoisomerase 2-binding protein 1
TP53	Cellular tumor antigen p53
TRAF1	TNF receptor-associated factor 1
TRCF	Transcriptional-repair coupling factor
TRX	Thioredoxin reductase
TTD	Trichothiodystrophy
USP7	Ubiquitin−specific−processing protease 7
TTDA	General transcription factor 2H subunit 5
TTDN1	Trichothiodystrophy non-phothosensitive 1
UVDE	UV damage endonuclease
UVER/UVDR	UVDE-mediated excision repair
UvrA/B/C	UvrABC system protein
UV-SS	UV-sensitive syndrome
WRN	Werner syndrome ATP-dependent helicase
XAB2	XPA−binding protein 2
XLF	Cernunos
XP	Xeroderma pigmentosum
XPC	Xeroderma pigmentosum group C
XRCC1/4	X-ray repair cross-complementing protein 1/4
